# Shame anxiety, stigma and clinical encounters

**DOI:** 10.1111/jep.13744

**Published:** 2022-07-28

**Authors:** Luna Dolezal

**Affiliations:** ^1^ Wellcome Centre for Cultures and Environments of Health University of Exeter Exeter UK

**Keywords:** health‐related stigma, medicine, phenomenology, shame anxiety, stigma, shame‐sensitive practice

## Abstract

Stigma has been associated with delays in seeking treatment, avoiding clinical encounters, prolonged risk of transmission, poor adherence to treatment, mental distress, mental ill health and an increased risk of the recurrence of health problems, among many other factors that negatively impact on health outcomes. While the burdens and consequences of stigma have long been recognized in the health literature, there remains some ambiguity about how stigma *is experienced* by individuals who live with it. The aim of this paper is to elucidate the phenomenology of stigma, or to describe how it is that stigma shows up in the first‐person experience of individuals who live with stigma and its burdens. Exploring the relationship between shame and stigma, I argue that shame anxiety, or the chronic anticipation of shame, best characterises the experience of living with a health‐related, or health‐relevant, stigma. Understanding the experiential features, or phenomenology, of shame anxiety will give healthcare professionals a greater sensitivity to stigma and its impacts in clinical settings and encounters. I will conclude by suggesting that ‘shame‐sensitive’ practice would be beneficial in healthcare.

## 1 INTRODUCTION

There is widespread agreement and acknowledgement that many health conditions, for example, mental disorders, obesity and infectious disease, are highly stigmatized and that this has far reaching consequences for those who live with them. As noted by Patrick Corrigan and David Penn, ‘Stigma's impact on a person's life may be as harmful as the direct effects of the disease’.^1^ Stigma has been associated with delays in seeking treatment, avoiding clinical encounters, prolonged risk of transmission, poor adherence to treatment, mental distress, mental ill health and an increased risk of the recurrence of health problems,^2^ among many other factors that negatively impact on health outcomes. As Norman Sartorius, psychiatrist and the former Director of the WHO's division of Mental Health, writes in a special section on ‘Stigma of Mental Illness’ in *The Lancet*, ‘stigma and discrimination are the most significant obstacles to the development of mental health care and to ensuring a life of quality to people suffering from mental illness’.^1^ What this means is that while an illness or health condition can cause physical discomfort, suffering and possibly even impairment, the stigma one experiences alongside the condition or illness can bring a whole host of additional social, psychological and health burdens. Stigma increases stress, decreases one's capacity to cope, affects mental health and may limit one's access to healthcare and health resources. Overall, the negative impacts of health‐related stigma are serious and directly impact on health and health outcomes.^3^

The burdens and consequences of stigma have long been recognized in health research, and there is a rich and growing literature that measures and categorizes different types of stigma and stigma‐related phenomena^3^—for example, felt stigma, internalized stigma, enacted stigma, anticipated stigma, stigma practices, stigma marking, and so forth. However, despite this rich conceptual landscape, for the non‐academic lay person or health professional, there remains some ambiguity more generally about how stigma *is experienced* by individuals who live with it in its various forms. The aim of this paper is to describe how it is that stigma shows up in the first‐person experience of individuals who live with stigma and its burdens in order to give healthcare professionals practical and concrete tools to negotiate stigma in clinical practice.

Exploring the relationship between shame and stigma, I argue that shame anxiety, or the chronic anticipation of shame, best characterises the first‐person experiential dimension of living with a health‐related, or health‐relevant, stigma. Designing useful tools or interventions for healthcare workers with respect to working with stigma, I argue, will be made possible by understanding the affective and emotional experiences that accompany living with stigma. For this reason, my focus in this article is to elucidate the experiential features, or phenomenology, of stigma‐related shame anxiety to give healthcare professionals a greater sensitivity to stigma and its impacts in clinical settings and encounters.

The methodology followed in this analysis is theoretical phenomenology, a mode of philosophical enquiry that endeavours to make rich descriptions of the structures of first‐person lived experience. In healthcare research, this method has been usefully employed, for example, to elucidate the lived experience of illness, a perspective which is often missing from, or at odds with, biomedical understandings of illness.^4^ Having a rich understanding how a medical phenomenon is experienced from a first‐person perspective, with respect to embodiment, sociality, engagement with the material environment, and one's world of possibilities, can yield important insights that can help to improve clinical encounters and holistic understandings of well‐being, illness, disease and suffering. My aim in this paper is to give an account, based on a theoretical and phenomenological perspective, regarding how the experience of shame, particularly shame anxiety, is central to understanding how stigma causes harm, especially in experiences of illness. I will conclude by suggesting that ‘shame‐sensitive’ practice would be beneficial in healthcare.

## 2 HEALTH‐RELATED STIGMA

The term ‘stigma’ is generally used to describe ‘the degrading marks that are affixed to particular bodies, people, conditions and places within humiliating social interactions’.^5^ Stigma is a socialized conception of what is disgraceful, unacceptable or abnormal within a particular social group, and being marked as stigmatized does not merely designate someone as different, but denotes them as profoundly discredited, denigrated, devalued and disgraced.^6^ Historically, stigmas used to be inflicted physically, through marking or branding bodies. This mark might be put on an individual's clothing, body or dwelling, for example, the scarlet letter, the shaved head, the yellow star, the dunce cap, the tattoo, the brand. In ancient Greek society, it was common to mark criminals with tattoos on the face, so that their offense was visible in a public way.^7^

In contemporary societies, stigmas are usually inflicted silently and invisibly through the social norms and political machinations of a dominant social group that mark out certain behaviours, physical characteristics or circumstances as unacceptable or deviant and, therefore, inferior.^8^ Stigma is a useful idea in healthcare. It helps make clear the social impact of illness,^9^ or, in other words, how the experience of an illness may coincide with a range of negative social events, such as discrimination, judgement, social exclusion, vilification, ostracism, labelling, status‐loss, prejudice, unfair treatment, among others.^10^

Despite a rich conceptual landscape with regard to stigma in health research, where various types of stigma are differentiated and theorized,^3^ in the day‐to‐day practice of healthcare, these nuances are often not present in lay understandings of stigma. The philosopher Phil Hutchinson notes that stigma ‘presents a puzzle for the researcher who might reasonably expect to find their phenomenon in the wild. Stigma is both everywhere and yet it can be difficult to find, at least without some productive or stipulative formal analytic work’.^11^ Stigma, as Hutchinson^11^ notes, is a category term, rather than something that is experienced directly (as we might experience the pain or discomfort associated with an illness). Stigma's simultaneous ubiquity and elusiveness results from the fact that it is a term used largely by researchers to designate and make sense of a wide range of phenomena, such as discrimination, unfair treatment, stereotyping and prejudice, while the individuals experiencing these phenomena are often ‘unlikely to employ the language of stigma’.^11^

As a category term used to designate ‘status‐degrading, discrediting and discriminatory interactions’,^11^ stigma itself is not something that is experienced *directly* by an individual. Instead, stigma is experienced *indirectly* through association with other events or experiences that one has in social or healthcare contexts, such discrimination, stereotyping or prejudice, and so forth. These sorts of experiences come to be understood *to be the result of the stigma* associated with one's illness, condition or circumstances. As a result, while the idea of what is termed ‘health‐related stigma’ has been discussed at length in academic literature, it remains ‘one of the most significant—and least understood—barriers to health promotion and disease prevention around the globe’.^12^ Stigma's enduring mystery in part lies in understanding how it shows up in lived experience. As Bennett et al.^13^ have noted recently in relation to HIV, ‘the mechanism by which stigma may cause distress remains largely unknown’. It is my contention that if one seeks to understand *the mechanisms through which* stigma related to health adds to the burden of illness, while also providing a concrete framework for healthcare practitioners to engage meaningfully with stigma to lessen its harms, then one must begin to investigate its emotional, personal and affective dimensions. For this reason, it is worth investigating shame.

## 3 SHAME AND STIGMA

Although a relationship between stigma and shame is widely acknowledged and it is often taken for granted that stigma causes negative self‐conscious emotions such as shame, humiliation and embarrassment, there remains a paucity of research that directly investigates how shame is experienced in relation to stigma. Shame is sometimes mentioned in relation to the *experienced* or *internalized* aspects of stigma, however, it is surprising to find that in much of the current literature regarding health‐related stigma, shame is not even mentioned.^9^, ^14^ When shame is discussed, there remains a lack of consensus about how shame and stigma are related or interact^13^, ^15^ (with the notable exception of Hutchinson and Dhairyawan's^16^ recent work that considers the connections between HIV stigma and shame). Nor is there much discussion of how shame related to stigma manifests, or shows up, in lived experience.

In contrast, the sociologist Graham Scambler directly considers how shame is related to stigma through considering the first‐person experience of stigma. Scambler^17^ discusses ‘felt stigma’ to designate how stigma *is experienced*. He suggests that ‘felt stigma’ has two parts: first, ‘the *shame* associated with’ being reduced to a condition (e.g., ‘being HIV‐positive’ or ‘being depressed’), and, second, ‘the *fear of encountering enacted stigma*’,^17^ where ‘enacted stigma’ is, for Scambler,^18^ synonymous with ‘shaming’. While Scambler's conceptual demarcations between various categories of stigma (e.g., ‘felt’ vs. ‘enacted’) are useful, for the purposes of this article, I leave them to one side to focus on what I see as Scambler's central claim: that there is a necessary relation between shame and the experience of stigma. He is of course, not the only person to suggest this, and many researchers end up using the terms ‘shame’ and ‘stigma’ almost interchangeably, taking for granted a connection between the *emotion* and *experience* of shame and the *social attribute* or *category* of stigma. Scambler's idea of shame being synonymous with ‘the fear of encountering enacted stigma’ I think is very useful as it highlights the structures of shame and shameful anticipation inherent to how stigma is *experienced*.

Shame is commonly characterized as a negative self‐conscious emotion; it is an experience that arises when we are concerned about how we are seen and judged by others. We feel shame when we are seen by another or others (whether they are present, imagined or simply a viewpoint that has been internalized) to be flawed in some crucial way, or when some part of our core self is perceived to be inadequate, inappropriate or immoral.^19^ As the phenomenologist Jean‐Paul Sartre notes, ‘shame … is the recognition of the fact that I am that object which the Other is looking at and judging’.^20^ During a shame experience, we can feel deeply and often irreparably flawed, unworthy and unlovable, and that our social position and our social bonds are under threat. If we understand shame in this way, then we see that stigmatized conditions, illnesses or circumtances inherently hold the potential for shame—stigma marks out someone as ‘degraded’, ‘damaged’, ‘abnormal’ or ‘less than’ or in other words ‘shameful’, in relation to prevailing social norms. Hence, living with stigma, one may experience *actual* shame as a result of one's condition, symptoms and perhaps on‐going treatment. At the same time, one may experience an *anticipated* shame as a result of worrying that one's condition will be revealed, noticed or discovered by others.

To give a concrete example of this phenomenon: a study by Brocq et al.^21^ of individuals living with obesity during COVID‐19 in the UK, reveals how stigma related to excess weight and obesity, which was intensified by the media and comments on social media during the pandemic, directly led to ‘feelings of shame’ and a perception ‘of being less of a priority than any other condition’. A consequence of heightened obesity stigma during the pandemic, they conclude, was the ‘avoidance of healthcare, probably worsening COVID‐19 outcomes’.^21^ They also discuss how the ‘fear’ of experiencing ‘stigma or shame’ led to the avoidance of certain public activities by individuals living with obesity, such as shopping and outdoor exercise.^21^ What this example demonstrates, is that living with stigma involves the experience of both *actual* and *anticipated* shame, and these can both impact directly on how an individual engages with healthcare and healthcare providers, along with health‐relevant behaviours and activities.

## 4 ANTICIPATED SHAME OR SHAME ANXIETY

Shame anxiety is defined by the theologian and shame theorist Stephen Pattison as an ‘anticipatory anxiety about the imminent threat of being exposed, humiliated, belittled or rejected’.^22^ Living with shame anxiety does not mean that one experiences shame constantly or continuously. Instead, in experiences of shame anxiety the threat of shame is more predominant and persistent. Shame anxiety is commonly characterised by the nagging and persistent *possibility of shame*, which, for the most part, is not necessarily experienced.^8^ Shame anxiety appears in first‐person experience as a corrosive, undermining and persistent anxiety about being objectified, judged, labelled and rejected by others. This shame anxiety ultimately can become connected to negative self‐beliefs and self‐conceptions; one can come to believe that the ‘core of their being [is] flawed, useless, despicable’.^23^

Instead of the searingly painful self‐consciousness that accompanies discrete episodes of actual shame, the experience of shame anxiety can render shame invisible, both to the self, who is experiencing it, and to others around them. Shame can be an intensely threatening and uncomfortable experience, which means that individuals will go to great lengths to avoid it. What this means is that shame may not present in experience, even when it is occurring. Instead, shame is anticipated and avoided, or bypassed altogether. The psychiatrist Donald Nathanson theorizes the ‘compass of shame’, where he describes the common defensive reactions that individuals deploy to avoid, or bypass, shame experiences. According to Nathanson, shame‐avoidance behaviours follow four common patterns: withdrawal, avoidance, attack other and attack self.^24^ Common defensive behaviours include a variety of different reactions, all of which are damaging both to oneself and to one's social bonds, such as anger, aggression, hostility, violence, narcissism, depression, perfectionism, apathy, withdrawal, avoidance, excessive deference, among others.^22^, ^24^ These reactions may happen automatically and unthinkingly, with individuals themselves not even realising that they are avoiding shame.

In addition to the compass of shame, shame avoidance can occur more intentionally through controlling one's actions and interactions, or one's behaviour and demeanour. Shame can also be avoided through steering clear of certain circumstances, encounters, situations or conversations. As Pattison notes about individuals who live with shame anxiety: ‘They live their lives trying to avoid occasions and relationships that might provoke painful shame experiences’.^22^ In this way, shame anxiety is not usually experienced *as* shame. Instead, it is an affective response, dominated by shame avoidance, that may include emotions such as fear, anxiety, stress or powerful impulses to hide, avoid or escape, along with negative feelings about the self, characterised by a sense of inadequacy, insecurity, defilement or deficiency in relation to others, particularly others who one feels may have the social power to pass judgement.^8^

Shame anxiety is difficult to identify and ‘diagnose’; it is an elusive experience that is often ‘disguised’ or ‘camouflaged’ by other experiences and feelings. As a result, shame anxiety often remains invisible. Individuals who experience shame anxiety do not live with constant shame. Instead, what they experience is not shame, but ‘what it costs them to keep from falling into shame’.^25^ As a result, what characterises the experience of shame anxiety is not enduring or repetitive experiences of shame but rather an atmosphere of anticipated shame, and a fear of shameful exposure, that leads to avoidance behaviours or experiences.

The queer theorist Kane Race describes the experience of shame anxiety eloquently in relation to his HIV‐status. In a semi‐autobiographical essay, he describes the experience of receiving an HIV diagnosis in 1996, a time when HIV was intensely stigmatized:

Soon after receiving my HIV diagnosis I made an appointment with a general practitioner with years of specialist experience in HIV care… ‘Don't tell your parents’, was the first piece of advice he offered me … I did hold off on telling my parents for some time … But living in constant fear of imminent exposure with a stigmatized, sexually humiliating condition takes its toll… It was only a matter of time before they discovered my dirty secret, I figured. In the narrative that dominated my parental imaginary, HIV operated as vindicating proof of sexual depravity: conclusive evidence of the wrongness of homosexuality and the foolish lifestyle I was leading. I imagined my parent's anger, disappointment, and deep shame on discovering that their only son had succumbed to the logical outcome of the homosexual lifestyle they had warned me against repeatedly. I had wasted my life, wasted everything, betrayed every hope they had invested in me. This apprehension of a wasted life filled me with a sense of impending catastrophe…. Intensely conscious of the shameful disappointment I had become, I lived in constant fear of public humiliation and unwanted exposure … [as a result of the] intense sexual shame that the possibility of having my HIV status outed to my parents unwittingly provoked in me.^26^

Race describes how a ‘constant fear' of exposure and of ‘impending catastrophe’ was intimately connected to the anticipation of a negative evaluation of the self through the eyes of an ‘other’, which was dominated by his ‘parental imaginary’.^8^ Race describes how he is *anticipating* shame: at any moment, humiliating exposure is possible; he may be judged for his ‘sexual depravity’ and subsequently rejected. The explicit anticipation of shame, or shame anxiety, related both to his sexuality and HIV‐status, both highly stigmatized attributes, comes to be a defining feature of his lived experience.

Using Kane Race's account as illustrative, if we follow Scambler and understand a key experiential component of stigma to be ‘the *fear of encountering enacted stigma*’,^17^ then shame anxiety can help elucidate how stigma manifests in day‐to‐day lived experience. If an individual lives with stigma, then they may live with the constant fear of feeling shame, of actively being shamed, or of shameful exposure—being made to feel that they are ‘less than’, that they are ‘unworthy’ that they are ‘contaminated’ or ‘disgraced’. These experiences commonly cause mental distress and harm. As a result, most of us will avoid shame, shaming and shameful exposure at all costs. As the philosopher Bonnie Mann notes, shame ‘produces necessity’, in so far as ‘one simply could not live and face one's community if one acted in a certain way, or did not act in a certain way’.^27^ In this way, experiences of shame and shame anxiety can challenge rationality and reason, where evidence shows that efforts to avoid shame can lead to individuals acting against their own best interests, or in other ‘unreasonable’ or ‘irrational’ ways.^28^ Hence, the ‘necessity’ to avoid shame can come at the cost of even harming or hurting oneself: for instance by not seeking medical help even when one is aware of a health issue.

## 5 SHAME ANXIETY IN HEALTHCARE

Shame has been a frequently unacknowledged and underemphasised aspect of clinical encounters within healthcare. Davidoff^29^ describes shame as the ‘elephant in the room’, puzzling over why shame is still reluctantly broached as a research topic, when there is evidence that ‘shame is a powerful force’ that has clear clinical impacts. Shame is, as he notes, something ‘so big and disturbing that we do not even see it, despite the fact that we keep bumping into it’.^29^ Shame is undoubtedly a common experience for patients. As Aaron Lazare^30^ notes in his seminal 1987 article on the subject, ‘Shame and Humiliation in the Medical Encounter’, ‘patients may experience physical or psychologic limitations as defects, inadequacies, or shortcomings … Treatments and their side effects may be potential sources of further shame and humiliation: mastectomies, the loss of hair and impotence are examples’. Clinical encounters, some argue, are inherently shame‐producing. As Salter and Hall^31^ argue, professional practice, like medicine and social care, are frequently ‘vectors of shame, humiliation and inequality’. Shame is very easily exacerbated and incited in the context of seeking help from professionals. Interactions with healthcare professionals can compound feelings of shame and shame anxiety, as these interactions often involve unequal power relationships, a fear of being judged, the scrutiny and exposure of one's potentially ‘shameful’ past, circumstances, coping behaviours, body, illnesses, mental health status along with other vulnerabilities.^32^ Exposing one's physical body and being subjected to physical exams, especially when the body is put into strange positions or sexual organs are examined, can be inherently shameful or humiliating.^33^ If one is already experiencing high levels of shame or shame anxiety because of living with stigma, then healthcare encounters, where shameful exposure can feel inevitable, may come to feel particularly threatening to one's sense of psychological safety, regardless of the attitude, intentions or demeanour of the healthcare professionals one encounters.

However, there is ample evidence that the shame is often implicitly and explicitly incited by healthcare professionals when they are confronted with stigmatized conditions. Healthcare professionals sometimes display outwardly judgemental attitudes, and reactions that make patients living with stigma feel they are ‘dirty or diseased’ or they have ‘done something bad or wrong’.^34^ In her article, ‘A dirty little secret: stigma, shame and hepatitis C in the health setting’, Jane Northrop^34^ analyses experiences of individuals living with Hepatitis C in clinical settings, where fearing ‘the disdain of healthcare workers’, fearing ‘being judged by hospital staff’ and being made to feel ‘uncomfortable’ were common experiences which frequently lead to ‘shame’ and ‘feelings of uncertainty and diminished worthiness’. These experiences are far from unique, where ‘medical shaming’, or the explicit and/or implicit shaming of individuals living with stigmatized conditions, such as mental illness, obesity and infectious disease, is reported as routine and commonplace in healthcare settings.^35^

In the context of seeking healthcare, it has been demonstrated that shame is a ‘potent treatment barrier’.^36^ There is ample evidence that the ‘necessity’ to avoid shame or shameful exposure can interfere with individuals accessing healthcare and negatively affect the quality of care they ultimately receive.^30^, ^33^, ^34^, ^37^ Hence, in the context of seeking or needing healthcare, individuals who are chronically anxious about shameful exposure because of living with stigma may avoid seeking help in the first place, may regularly miss appointments, may avoid disclosing honest details about symptoms, lifestyle or circumstances, may fail to follow through with treatments and may conceal diagnoses and coping behaviours from friends, family and professionals.^16^, ^37^ In the case of HIV, a highly stigmatized illness, Hutchinson and Dhairyawan^16^ highlight various ways that shame can negatively impact on the treatment of HIV, which includes: nondisclosure to clinicians about sexual histories, not engaging with care, not presenting for testing, not disclosing HIV status to new sexual partners, and causing a psychological burden where living with HIV becomes a far more negative experience than it needs to be.

In healthcare contexts, stigma is often recognized to be related to illness or associated with one's health condition or body. Many illnesses and conditions, such as obesity, HIV and lung cancer, are commonly stigmatized for their association with purportedly ‘negative’ lifestyle habits and the idea that they are ‘self‐inflicted’. Infectious diseases are also heavily stigmatized because of fears around contamination and infection. And many other conditions are stigmatized merely because they deviate from widespread standards regarding what is ‘good’, ‘proper’, ‘healthy’ or ‘acceptable’. However, it is important to note that stigma and shame in the clinic may not at all be health‐related, but nonetheless be health‐relevant. Stigma associated with low literacy levels, poverty, social deprivation, food insecurity, homelessness, race, ethnicity, immigration status, criminal justice, sexual violence, domestic abuse or other traumas, may manifest in healthcare settings as shame anxiety, a fear of one's shameful secret, circumstances or personal history being discovered by a healthcare professional. It seems clear that being attuned to experiences of shame and shame anxiety, along with the common tactics or strategies deployed to avoid shame and shameful exposure (e.g., avoidance, lying, withdrawal, nondisclosure), becomes central to successful healthcare, and in fact central to getting individuals to even seek help and engage with services.

## 6 SHAME‐SENSITIVE PRACTICE IN HEALTHCARE

Ensuring that healthcare professionals are aware of, and have competence to deal with, shame and shame dynamics can improve the quality of the delivery of healthcare and health services. As Lazare^30^ suggest, ‘physicians should assume that any disease (and treatment) can be a shame‐inducing event which then interacts with a patients individual vulnerabilities’. Focusing healthcare interactions through a ‘shame lens’, particularly when considering stigma, will reveal significant affective dynamics that may otherwise be occluded and which have the potential to interfere with successful care.^32^ Shame‐sensitive practice involves *acknowledging shame*, *avoiding shaming* and *addressing* shame.^32^ This means not only attempts at minimizing unhealthy shame, thereby reducing the potentially damaging and ‘debilitating effects of shame’,^39^ but also an awareness of shame dynamics, where healthcare practitioners are more attuned to bypassed, deflected or invisibilised shame and its consequences, while also being alert to ways that shame and shaming may be produced through interpersonal dynamics along with organizational practices and policies.^32^ In this way, shame‐sensitive practice is integrated at the interpersonal level—in interactions between practitioner colleagues, and between practitioners and patients—and also at the organizational and policy levels—with an understanding of how institutional structures, practices and policy decisions can exacerbate or create conditions for shame and shaming.

At a basic level, shame‐sensitive practice starts with healthcare professionals having ‘shame competence’.^32^ Shame competence involves healthcare practitioners having a theoretical and practical understanding of shame and shame dynamics.^39^ Practitioners must understand what shame is and how it is commonly incited. They must be aware of, and able to identify, behaviours that are used to cope with shame, and understand the common ways that shame is hidden and concealed. Practitioners must also be aware of shame dynamics, how shame circulates interpersonally, and develop on‐going competence in identifying their own shame and its effects on their thinking, actions and behaviour within professional practice, particularly to avoid the ‘inadvertent humiliation of a patient’ through discharging their own shame.^30^ Individual shame competence must be developed within a framework of organizational shame competence. This involves the fostering of emotional intelligence within workplaces and professional practice, where speaking about and understanding emotions, and their effects, within professional practice becomes commonplace.^32^, ^38^ In particular, the taboo regarding shame, and shameful or stigmatized states and experiences, must be directly addressed and discussed openly.

In addition to shame competence, healthcare professionals must be trained to avoid explicit and implicit shaming. In interpersonal encounters with patients, healthcare practitioners must be alert to the potential for implicit shaming in interactions.^32^ Practitioners must continuously assess, how the language they use, their demeanour, questioning style, emotional expression and other interpersonal dynamics may inadvertently produce a shame response.^32^, ^34^, ^40^ Furthermore, practitioners must consider interpersonal dynamics, based on gender, race, ethnicity, language‐spoken, disability, age, religious identification, along with other factors in particular situations. Practitioners should always avoid stereotyping, labelling and other dehumanising and stigmatizing ways of engaging with individuals. To avoid inciting shame, it is imperative to prioritise relational practice. Practitioners must remain responsive to individuals and their unique circumstances and to genuinely acknowledge distress. Individual practitioners' ability to be ‘shame sensitive’ is scaffolded by a range of other factors, such as organizational structures that are in place to support shame competence and to help practitioners foster shame resilience, while also providing the resources to combat and address the systemic causes of shame and stigma.^32^, ^38^

**Table:** 

Principles for shame‐sensitive practice	
Acknowledge shame	− Develop shame competence
Avoid shaming	− Avoid implicit and explicit shaming
Address shame	− Foster shame resilience and combat and address systemic causes of shame and stigma

*Source*: Dolezal and Gibson.^32^

## 7 CONCLUSION

It is understandable that shame is often left out of discussions of stigma because shame itself is shameful, and identifying someone as experiencing shame can cause distress and be disempowering and damaging. Shame, after all, implies that one is flawed, defective and at fault. However, shame is by no means merely a private emotional event. It is important to understand shame as part of a ‘nexus’, which spans individual experiences of ‘felt shame’ but also necessarily includes shared socio‐political norms, along with broader power dynaimcs.^41^ It is also important to understand that social power is central to shaming, where those with more social power can more readily shame those with less. Being cognizant of the power one wields in a healthcare setting, and the affective consequences this power may have, is imperative as shame can occur very easily in a healthcare setting where power imbalances and the scrutiny and judgement of bodies and behaviours are the default characteristics of clinical encounters. Acknowledging shame in healthcare encounters has the potential to illuminate behaviours, attitudes and actions which may get in the way of successful care, particularly when considering health‐related or health‐relevant stigma.

## CONFLICT OF INTEREST

The author declares no conflict of interest.

## Data Availability

Data sharing not applicable to this article as no data sets were generated or analysed during the current study.
